# Analytical investigation of multi-layered rollable displays considering nonlinear elastic adhesive interfaces

**DOI:** 10.1038/s41598-023-31936-7

**Published:** 2023-04-07

**Authors:** Sang Hyun Han, Jun Hyuk Shin, Su Seok Choi

**Affiliations:** Department of Electrical Engineering, Pohang University of Science and Technology (POSTECH), 77 Cheongam-Ro, Nam-Gu, Pohang, Gyeongbuk 37673 Korea

**Keywords:** Electrical and electronic engineering, Electronic devices

## Abstract

To design the multilayered structures of reliable rollable displays, finite element method (FEM) investigations are conducted at various rolling conditions. Given that the optically clear adhesive (OCA) is the only flexible component and interfacial layer that plays an important role in allowing flexibility in rollable displays, we investigated its nonlinear elastic properties in detail. Hereto, FEM of rollable displays have been limited and inaccurate because OCA has been assumed to be a linear elastic material. In addition, despite the fact that rolling deformation exhibits complex bending characteristics, unlike folding, the mechanical behaviors over the entire area of rollable displays at all positions have not yet been addressed. In this study, we describe the dynamic and mechanical characteristics of rollable displays at all positions considering the hyperelastic and viscoelastic properties of OCA. The maximum normal strain of the rollable displays was applied about 0.98%, and the maximum shear strain of the OCA was shown to be around 720%. To assess the stability of the rollable displays, normal and yield strains were compared to each layer and investigated. Consequently, mechanical modeling of the rollable displays was conducted and stable rolling behaviors that did not cause permanent deformation were investigated.

## Introduction

As the importance of display form factors has recently increased, research interest in flexible display technology has also been growing rapidly. From the viewpoint of user experience (UX), the evolution of flexible displays can be classified in various categories ranging from simple flexible displays (e.g., curved) to dynamic screen displays^1^. The dynamic screen and size deformable displays provide enhanced UX freedom in using displays by enabling shape screen changes in response to user demands. Ideally, a fully stretchable display is desired for this shape deformable display^2–4^. However, ideal and also practically available stretchable displays require additional technical innovations despite the intensively growing research interest. Moreover, the organic light emitting diode (OLED) display is currently the major flexible display technology available for practical purposes, but this technology is not fully ready for use in stretchable displays. Therefore, screen with extendable display manipulating OLED displays, such as foldable and rollable types, have also attracted research interests, particularly for practical purposes, which are envisaged to be prevalent until stretchable displays are ready and available.

Foldable displays constitute an early technological type of extendable displays with limited and simple screen extension characteristics, and are now commercially available. In contrast to the limited screen deformation in foldable displays, rollable displays should experience various dynamic mechanical stress over the entire display structure to obtain fully rolled display shapes. However, full and on-demand, extendable screen displays, referred to as “rollable displays,” are still under research investigation, in spite of the fact that the first rapid-prototype concept display was introduced approximately 10 years ago^5,6^. Although large-sized rollable televisions (TVs) with low-mechanical deformation characteristics and simple stack structures have been developed, more rollable displays are desired in small sized display applications. However, accomplishing this task is still challenging due to the operations in adverse rolling stress conditions with multilayered structure and small rolling radius with large stress.

The improvement of flexibility against mechanical shape deformation can be achieved by introducing a soft material, or by reducing the overall thickness of the device^7^. These approaches can be found in various simple, stacked flexible applications, such as in simple, transistors^8,9^, touch panels^10^, solar cells^11^, thin-film batteries^12^, photonic devices^13^, piezoelectric devices^14–16^, and other electronics^17–19^. However, flexible displays using OLED, such as rollable displays, are problematic owing to the more complex multilayered structures composed of multiple components. Appropriate stacking of multiple components, such as in cover window, circular polarizer, active matrix OLED (AMOLED), and back film is required to achieve stability in full rolling deformation conditions. This results in technical difficulties associated with the implementation of reliable displays without loss of display working performances in mechanical, optical, and electrical properties. Therefore, fully rollable multistacked OLED displays require comprehensive and careful considerations of material components and multistack display structural models in adverse, dynamic, mechanical deformation rolling conditions.

The optically clear adhesive (OCA) is the polymeric optical interface layer used to enable multistacked OLED to achieve optical clarity and interfacial bonding adhesions between rigid functional layers, such as those in cover window, circular polarizer, AMOLED, and back film^20–23^. In addition to the optical and adhesion benefits, OCA layers can be the key contributions for improved rollable displays. OCA can directly affect the mechanical properties of the display structures when subjected to bending or rolling deformations.

Recently, the most effective way to achieve flexibility is to modify the thickness or modulus of each layer of a flexible device, including OCA, to manage neutral planes close to rigid functional layers that are susceptible to stress^24–26^. Further, multi neutral planes can be preferred compared with the single neutral plane for improved mechanical stability in complex shape deformation conditions in multistacked layer cases^27–30^. Therefore, the OCA properties have to be understood to achieve improved, multi neutral planal designs, and rollable displays have to be implemented based on considerations of the multiple interfacial OCA layers between multistacked components^31,32^.

Conversely, finite element method (FEM) analysis can be considered to analyze numerically the mechanical deformation behaviors of neutral planes and shape deformations of multistacked OLED displays^33–45^. However, most previous studies were focused on simple bending of foldable displays rather than on complex shape deformation behaviors of rollable displays. Fully rollable displays experience complex and adverse deformations over the entire display area compared with foldable displays. Therefore, more careful and comprehensive investigations of rolling deformations and multi neutral plane effects of OCA layers are required for rollable displays. A few previous studies presented the mechanical deformation behaviors of rollable displays based on the use of strain distributions and OCA considerations^44,45^. However, in these previous studies on foldable and rollable displays, the soft OCA layers were simply considered as linear rather than nonlinear elastic materials. Therefore, these studies were limited in their capacities to achieve a reliable understanding of the complex shape deformation of FEM results for rollable displays. To implement realistic analysis, the soft OCA interfaces should be considered as nonlinear elastic materials with hyperelastic and viscoelastic properties. The characteristics of these nonlinear elastic materials show differences from those of the linear stress–strain elastic materials. From a practical point-of-view, the stress–strain curve shape of OCA is not linear in large deformations of rollable displays; moreover, the stress relaxation as a function of time during the mechanical deformation process should also be considered. Therefore, further considerations of the hyperelastic and viscoelastic properties of OCA are required to facilitate the understanding of large deformations of fully rollable displays.

In this study, an improved mechanical FEM analysis of rollable displays was suggested based on considerations of the hyperelastic and viscoelastic properties of OCA. Information on OCA based on experimental data was used to consider the properties of OCA in FEM. Further, numerical evaluations of all the OCA layer effects on multistacked and fully rollable deformations were investigated by varying the OCA modulus properties. The normal strain distribution of the rigid functional layers and the shear strain distribution of the OCAs during the dynamic rolling deformation processes were also examined. A comprehensive analysis with realistic modeling of the OCA interface and complex rolling deformations was conducted to understand in-depth, fully extendable rollable displays.

## Methods

**Figure 1:**
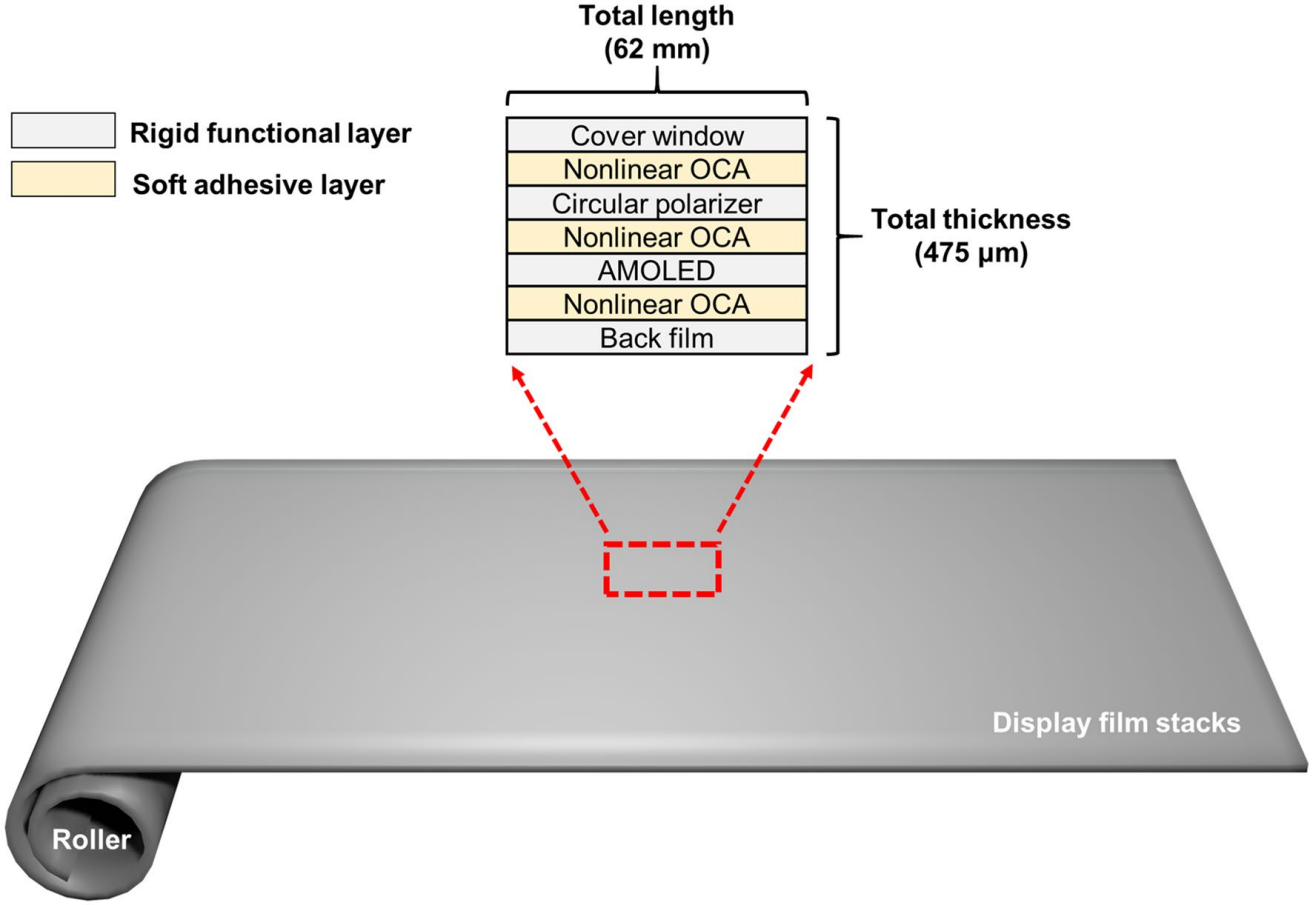
Schematic of Rollable display structure.

Figure 1 shows a schematic of a rollable display. Rollable displays are divided into the roller part and display stacks, including the multifunctional component layers. In this mechanical structure, the roller was designed so that the display can be rolled at a certain curvature. Additionally, the display film stacks are mechanical structures in which all components of the display are stacked. Film stacks are classified into rigid functional layers and soft adhesive layers. Rigid functional layers, such as the cover window, circular polarizer, active matrix oled (AMOLED), and back film, refer to hard layers. Conversely, soft adhesive layers, such as the OCA, refers to the very soft layers that provide flexibility so that the rigid functional layer can be rolled. Modeling of the OCA using a FEM has a significant impact on the reliability of a flexible display.

Previous FEM studies on rollable displays^44,45^ assumed and considered the OCA as a linear and fully elastic material used to reduce the complexity of FEM. However, in practice, OCA is a nonlinear elastic material, and its material properties that can describe nonlinear behaviors must be considered in FEM. The nonlinear elastic properties of OCA are hyperelasticity and viscoelasticity. Hyperelasticity refers to a nonlinear elastic response used to describe the stress–strain relationship for materials with large deformation behaviors, such as rubbers and elastomers. Therefore, hyperelasticity can represent the OCA’s behavior accurately compared with the Young’s modulus and the stress–strain relation of linear materials. Viscoelasticity refers to the material responses, including elasticity and viscosity. Elasticity is based on instantaneous relationships between force and deformation, and viscosity has time lags in which the relationship between force and deformation changes over time. In these viscoelastic properties, the stress relaxation effect that was not induced in linear elastic OCA, also occurs. Therefore, both hyperelasticity and viscoelasticity are material properties of OCA that need to be considered to obtain reliable FEM, rollable display analysis outcomes.

The cross-section of the bent film stacks contains many useful information for understanding the mechanical behaviors of the rollable display. First, the results for the normal strain distribution of rigid functional layers can be calculated. This normal strain distribution is necessary to evaluate whether each rigid functional layer is tensiled or compressed at a particular location; moreover, comparison of the normal strain of the FEM result and yield strain of each layer material is possible to evaluate whether the material is in the elastic or plastic region. Additionally, it is very easy to determine whether a neutral plane is created. Second, it is possible to measure the normal strain distribution of the rigid functional layer and the shear strain of the OCA. The shear strain of OCA is an indicator that determines how well the OCA (inserted between the rigid functional layers) works as a stress buffer layer^23,44^.

### Mechanical boundary conditions

**Figure 2:**
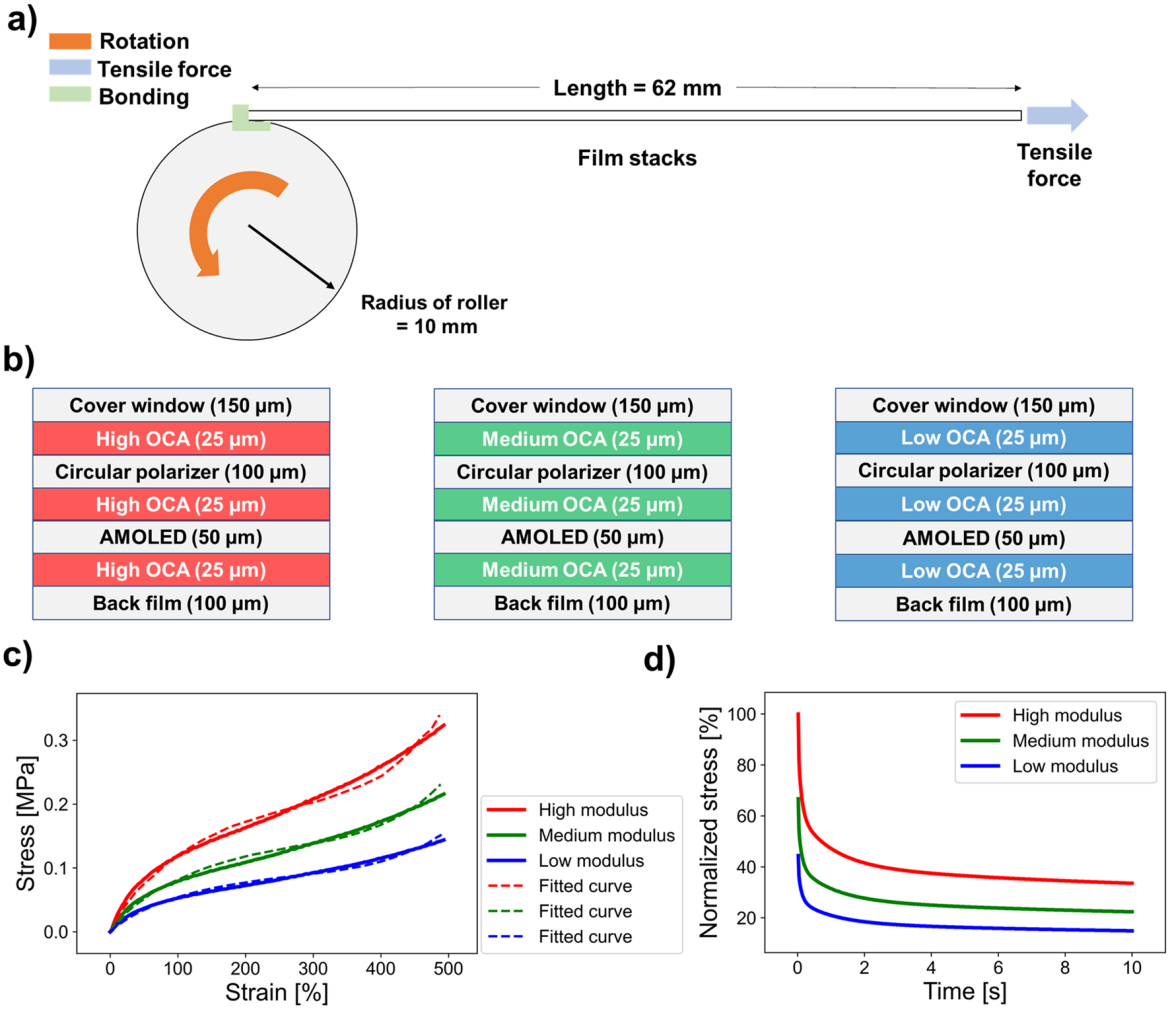
(**a**) Mechanical structure and boundary conditions of rollable displays. (**b**) Schematic of display film stack modules according to the modulus conditions of OCA. (**c**) OCA’s stress–strain curves with high, medium, and low modulus and fitted curves based on the use of the hyperelastic constitutive model. (**d**) Stress relaxation of OCA with high, medium, and low modulus at constant tensile strain.

Figure 2a illustrates the structure of rollable display and mechanical boundary conditions. We used the ANSYS Workbench 2020 R1 for FEM analysis of the rollable display. The geometrical model was selected as a two-dimensional (2D) plane-strain model. It was considered reasonable to choose an analytical model for the display film stacks with 2D rather than three-dimensional (3D) shapes because rollable displays have a very long and constant shape in the direction of thickness. This reduced the complexity of the model and the required processing time. The roller had a circular shape and a radius equal to 10 mm. Film stacks consisted of a cover window (150 μm), OCA (25 μm), circular polarizer (100 μm), OCA (25 μm), AMOLED (50 μm), OCA (25 μm), and a back film (100 μm) with a total length and thickness equal to 62 mm and 475 μm, respectively. The FEM conditions of the rollable display configuration and position settings of multistacks and mesh configuration are described in Supplementary Fig. S1. The Young’s modulus and Poisson's ratio values of the rigid functional layers in the film stack design are listed in Table 1. The rotation of the roller, pulling of the film stacks, and bonding of the bottom surfaces of the film stacks to the top surface of the roller are the three main boundary conditions that enable deformation of the rollable display. Rolling deformation was possible without wrinkling the film stacks when these three boundary conditions matched. We controlled the angular velocity to achieve a rolling deformation that rotated 360° in 5 s. In order to obtain the applied normal strain in the multi stacked components by bending, we adjusted the roller’s friction coefficient to be almost zero whereas 0.5 N of tensile force was set to avoid any warpage during rolling deformation process.

**Table 1:** Young’s modulus and Poisson’s ratio values for each rigid functional layer in the rollable display film stacks.

Layer	Young’s modulus (MPa)	Poisson’s ratio
Cover window	8000	0.3
Circular polarizer	20,000	0.35
AMOLED	2500	0.35
Back film	2500	0.3

### OCA modeling

Note that previous FEM studies considering the hyperelastic and viscoelastic properties of OCA were performed only on foldable displays rather than large variations of rollable displays^38,39,41^. Therefore, it was necessary to evaluate the OCA material with nonlinear characteristics before examining the mechanical behavior of the rollable display. We also attempted to assess the impact of the modulus of OCA on rollable displays. The film stacks shown in Fig. 2b were modeled by altering only the OCA modulus, while all the other material properties were unaltered. Herein, three different modulus conditions of OCA with high, medium and soft stiffness were considered for FEM analysis of rollable display. The stress–strain curves for each OCA are shown in Fig. 2c along with the fitted results based on the use of the hyperelastic constitutive model^23^. The fitting errors between the stress–strain and fitted curves were very small. Depending on the strain range of the material to be fitted and the shape of the stress–strain curve, users can select conveniently appropriate constitutive models, such as the Neo-Hookean, Yeoh, and Ogden^46,47^. Among them, we chose the Yeoh model as the constitutive model that best fitted our OCA stress–strain curve, as shown in Supplementary Fig. S2. The Yeoh model was also used to obtain the shear stress–strain calculation curve from the tensile stress–strain curve, which are shown in Supplementary Fig. S3. We described this model using the strain energy potential. For incompressible materials, such as OCA, the Yeoh model’s strain energy potential is

1 $$W = { }\mathop \sum \limits_{i = 1}^{N} C_{i0} \left( {I_{1} - 3} \right)^{i}$$

where C_i0_ are the material parameters, and I_1_ is the first strain invariant of the Cauchy–Green deformation tensor. The fitting parameters of the Yeoh model for each OCA are listed in Table 2. In addition to the hyperelastic properties of OCA, the viscoelastic behavior of each OCA was evaluated based on stress relaxation tests. Each OCA was stretched with a constant deformation for 10 s. The normalized stress of each OCA was observed over time, and the results are shown in Fig. 2d (please also refer to the Supplementary Video S1 on stress relaxations of the linear and nonlinear elastic OCAs). If OCA is assumed to be an elastic (e.g., a spring) rather than a viscoelastic material, there would be no decrease in stress over time. Therefore, the realistic properties of OCA, hyperelasticity, and viscoelasticity, must be modeled to improve the reliability of mechanical FEM of rollable displays.

**Table 2:** Hyperelastic fitting parameters for optically clear adhesives at low-, medium-, and high-modulus values.

Parameter	Low modulus	Medium modulus	High modulus
C_10_ [Pa]	15,021	22,532	33,797
C_20_ [Pa]	− 176.22	− 264.34	− 396.5
C_30_ [Pa]	2.8367	4.2551	6.3827

## Results and discussion

### Rolling deformation conditions

**Figure 3:**
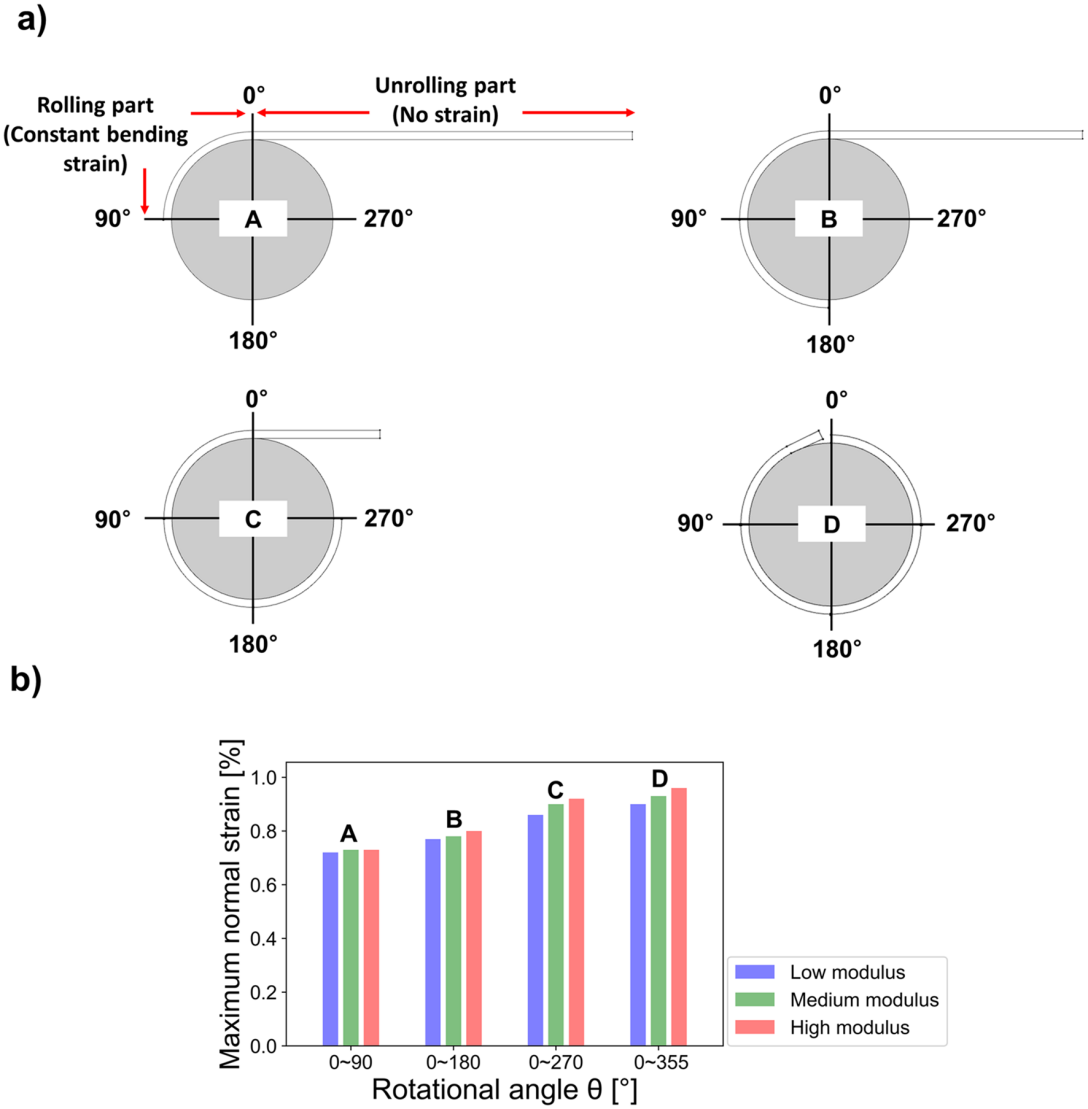
(**a**) Rigid functional layers with respect to the rolling deformations of 90°(A), 180°(B), 270°(C), and 360°(D). (**b**) Maximum normal strain results according to the rotation angle of rolling deformation based on the use of OCA materials with different modulus.

Calculation of the normal strain applied to the display in accordance with the rotation angle is a straightforward technique to understand the mechanical behavior of a rollable display. It is simple and can be used to determine the rolling deformation conditions where most susceptible to strain by using this approach. Furthermore, to evaluate whether the layer in the most vulnerable position is undergoing permanent deformation, it is crucial to compare the yield strain of each rigid functional layer with the normal strain derived from the FEM results. This makes it possible to determine whether the display would function safely within the elastic region. Depending on rotation angle, a rolling part where the rollable display is bent and an unrolling part where is not bent can be distinguished, as shown in Fig. 3a. Because the entire thickness of the display film stacks was relatively small compared with the radius of the roller, it was difficult to observe rolling deformation in practice. Therefore, we considered the thickness of the film stacks and depicted it schematically. Here, the rolling states A, B, C, and D correspond to rotations and deformations of 90°, 180°, 270°, and 360° in the rollable display, respectively. When the rotation angle was determined, it was found that the strain of unrolling part was almost zero, whereas the rolling part had a constant normal strain in same value. Figure 3b demonstrates that the normal strain of the rigid functional layers increases as a function of the OCA modulus and rotational angle.

First, the maximum normal strain at a rotational angle equal to 90°(A) had a comparatively low value. The normal strain of each rigid functional layer contained in the rolling part was almost same. For example, the normal strain at the cover window was found to be same value at positions of rotational angles between 30° and 80°. Also, the constant trend of normal strain for each rigid functional layer in the rolling part under 180°(B) rolling condition was also same for the rotation angles from 0° and 180°. However, note that the maximum normal strain value in the rolling part under 180°(B) rotation was higher than the normal strain under 90°(A) rotation condition. The constant normal strain but increase of maximum normal strain in rolling part was also in same tendencies at 270°(C) and 360°(D) rotations as well. For 360° (D) rolling deformation, the maximum normal strain was analyzed at 355° condition since there was a structural problem at 360° where the normal strain of rigid functional layers radically changed due to the aforementioned bonding boundary conditions in FEM condition.

Notably, the cover window, located at the top of the rigid functional layers, was found to be the place of all maximal normal strain. A mechanism has been described to understand the relationship between the rotation angle and the maximum normal strain in order to further explore the rolling deformation behavior. Because the height of the neutral axis changes as a function of rolling angle, the maximum normal strain of the rigid functional layers was also increased. From the viewpoint of bending mechanics, the normal strain should be determined by the radius of the roller, the height of the neutral axis, and the thickness of each layer^45^. On the other hand, the normal strain is believed to be a function of the height of the neutral axis because the roller's radius and the thickness of each layer are constant during rolling deformation process. Therefore, it could be confirmed that the height of the neutral axis of each cover window should decrease and resulted in eventually higher strains when the rotation angle was increased. Consequently, it can be believed that an increase in the rotational angle would lead the normal strain increase during rolling deformations. However, it was found that the normal strain at rigid functional layers also increased as a function of OCA modulus independent of the rotational angle. Shortly, it can be confirmed that the cover window with a high modulus OCA under 360° rolling strain condition was most vulnerable layer and condition to normal strain. Under these conditions, the highest normal strain of the cover window was approximately 0.98%. This result was less than the yield strain of the cover window, which was equal to 1.2%^44^. Therefore, we could verify that even the highest possible normal strain induced owing to rolling deformation was in the elastic region. Additionally, the yield strains of all these rigid functional layers was greater than 1%, and the maximum strain of the circular polarizer, AMOLED, and back film did not exceed 1%. Therefore, it can be assumed that the rigid functional layers were all placed in an elastic region that avoided permanent deformation.

### Characteristics of normal strain distributions using conventional linear elastic OCA model

**Figure 4:**
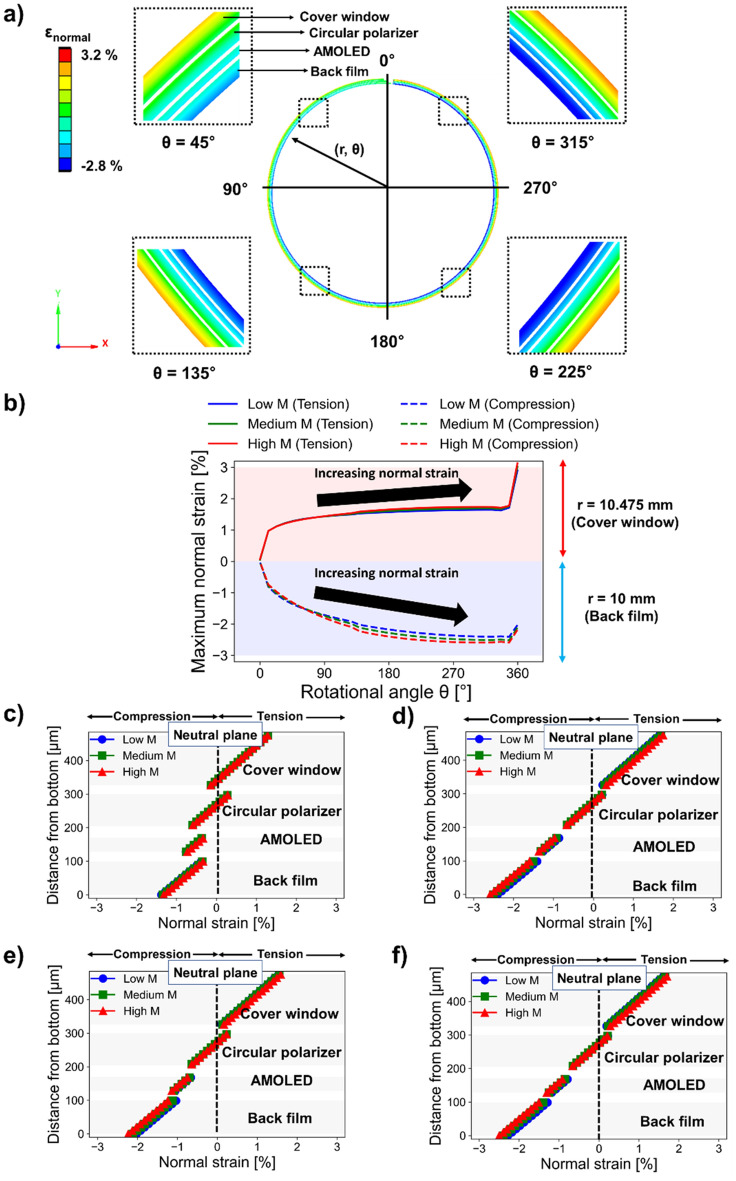
Conventional analysis of multi-layers rollable display using simple linear elastic OCA model. (**a**) FEM images showing the normal strain distributions with a mid-modulus OCA value. (**b**) Measurements of maximum tensile and compressive strains depending on the rotational angle. (**c**–**f**) Normal strain distributions at the cross-section of rigid functional layers at θ = 45°, 315°, 135°, and 225°.

First, to compare the conventional approach in previous studies^44,45^, simple linear elastic model OCA for multi-layered rollable display was examined in Fig. 4. Since the OCA of the linear elastic model was assumed to be a stiff material without consideration of viscoelasticity, shear deformation of OCA was small between rigid functional layers. The polar coordinate system (*r*, *θ*) was used to represent the position of the rigid functional layers, where *r* is the distance from the center of the roller to a specific position, and θ is the angle rotated counterclockwise about the y-axis. The normal strain distribution for θ = 45°, 135°, 225°, and 315° in a 360° rolling deformation was demonstrated in Fig. 4a. As a result, it was found that the multi neutral planes effect by using of OCA layers was not sufficiently investigated using simple linear elastic model of OCA. Figure 4b shows the maximum normal strain according to the rotation angle of the cover window and back film. Low M, Medium M, and High M denote low, medium, and high modulus of OCA, respectively. As the yield strain of the cover window is relatively low in susceptible to bending strain, we investigated in focused on the cover window rather than the back film. The yield strain at the cover window was 1.2%, as reported in a previous study of a rollable display by a display panel manufacturer^44^. It was also found that the maximum normal strain using the linear elastic model was increased up to 3.2%. However, the result of 3.2% near 360° was assumed to be an outlier caused by the bonding boundary condition between the roller and the display stack. It should be noted that the normal strain of the cover window exceeding yield strain of 1.2% was found earlier at rotation angles starting 35°. Therefore, nevertheless multi-layer OCAs were utilized for relieving strain, undesirable plastic deformation of the cover window was expected in this FEM analysis method based on simple linear elastic model. The normal strain distributions over the multi-layered rollable display for various cross-section at θ = 45°, 135°, 225°, and 315° are examined as shown in Fig. 4c–f. As a result, it was found that simple single neutral plane, which is also not desirable in multi-layered rollable display, rather than proper multi-neutral planes was created as the rotation angle increases. Therefore, it can be assumed that the FEM using linear elastic OCA models exhibits to result in incorrect and vulnerable rolling deformations analysis for rollable displays. Consequently, it is believed that linear elastic OCA models without considering the realistic characteristics of OCA are insufficient to investigate reliable rollable display analysis.

### Characteristics of normal strain distributions based on non-linear elastic OCA model

**Figure 5:**
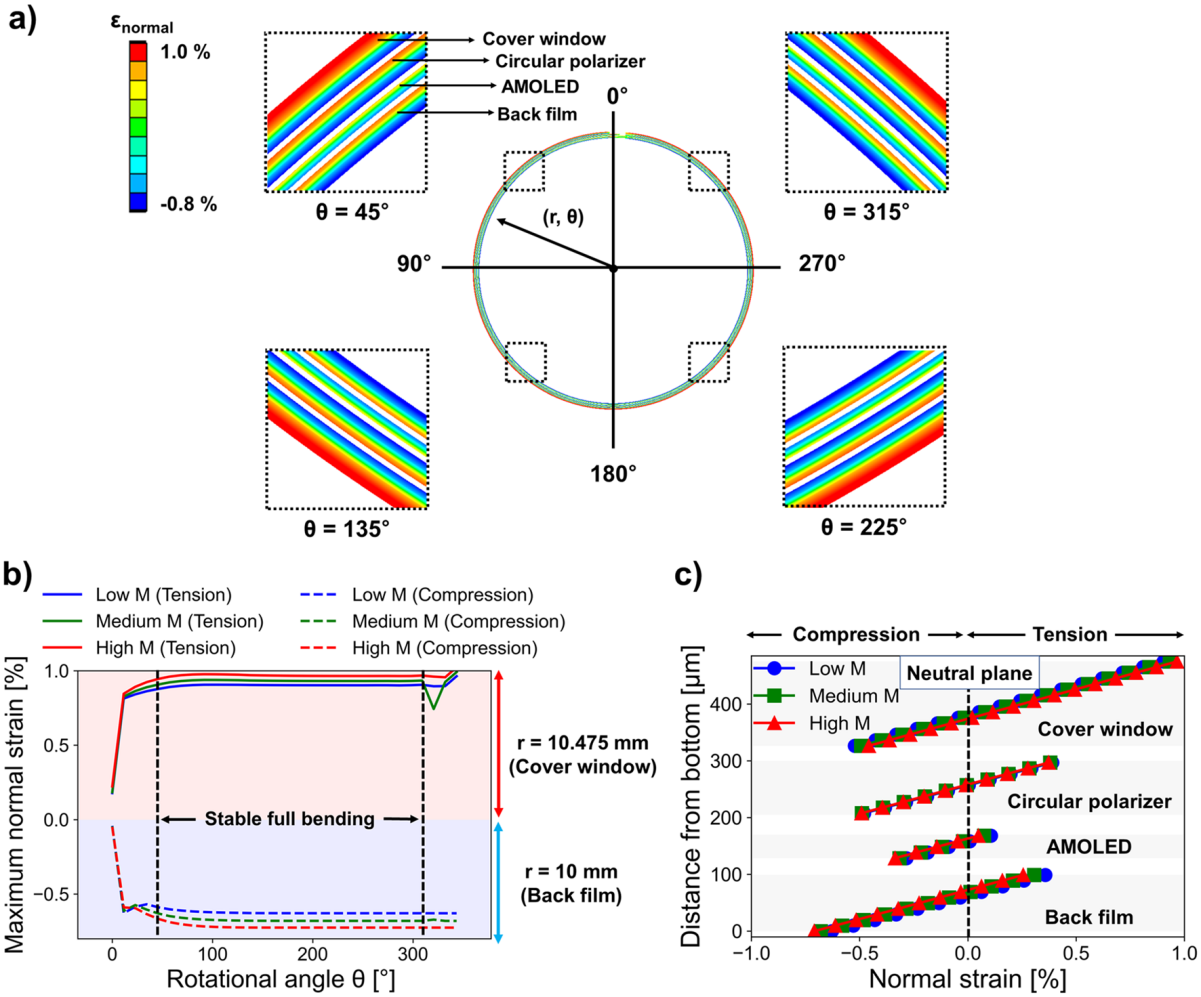
Finite element method (FEM) results on the behaviors of rigid functional layers in rolling deformation. (**a**) FEM images showing the normal strain distributions based on the use of OCA with a mid-modulus value. (**b**) Measurements of maximum tensile and compressive strains depending on the rotational angle. (**c**) Normal strain distributions at the cross-section of rigid functional layers at θ = 180°.

In contrast to the linear elastic OCA model, an improved analysis of multi layered rollable displays considering the realistic (nonlinear) properties of OCA was conducted. Considering a maximum rolling deformation equal to 360°, which was the most susceptible to normal strain, Fig. 5 shows the mechanical behaviors of rigid functional layers whose strain response outcomes were reduced by OCA. Herein, we were interested in the precise measurements of the normal strain of each rigid functional layer that contributed to the operation of the display film stack.

First, the FEM result for a 360° rolling deformation is illustrated visually in Fig. 5a. The OCA layer was visually hidden from the FEM results so that the results of each rigid functional layer can be compared. Normal strain distributions were demonstrated at different polar angles of θ = 45°, 135°, 180°, 225°, and 315°, respectively, to visualize the rigid functional layers exhibit almost same normal strain distributions at almost all locations. The quantitative normal strain distribution at all rotation angles measured from FEM analysis are shown in Fig. 5b. Here, we calculated the maximum tensile strain and maximum compressive strain in separate to understand the mechanical behavior of the rolling display in more detail. In rigid functional layers, the cover window experienced the highest tensile strain at r = 10.475 mm, whereas the back film had the highest compressive strain at r = 10 mm in polar coordinate. In addition, we were able to examine the findings of three sections’ normal strain distribution depending on the rotation angle, Fig. 5b. The first section, at the right end of the display film stack (left positioned section in the Fig. 5b) can be defined as the observing polar angle between 0° and 45° under 360° rolling deformation. Because the rigid functional layers were not entirely bent by the roller, this section exhibited a relatively low maximum normal strain. In contrast, the rigid functional layers were fully bent in the second section (middle positioned section in the Fig. 5b), where the rotation angles were between 45° and 315°. The maximum normal strain was constant because the neutral plane height, the thickness of each layer, and the roller's radius were all constant in this section^45^. We call this section as the stable full bending section in this report. In third section (right positioned section in the Fig. 5b), where the rotation angle exceeds 315°, the radical change of the maximum normal strain was inevitable due to the bonding boundary effect at 360° rolling condition in FEM setting. Therefore, we investigated the stable full bending section (second section) to analyze the performance of main normal strain distribution in rollable displays. In this stable full bending section, the tensile or compressive strains increased when the OCA of the rigid modulus was utilized.

In order to better understand the stable full bending section, we further investigated the cross-section of the stable full bending section at θ = 180° polar angle position as shown in Fig. 5c. As a result, it was clearly observed that each layer of the rigid functional layers created multi neutral planes. Therefore, it can be assumed that the strain relieving multi neutral planes of OCAs inserted between the rigid functional layers of rollable display were created properly. In addition, we examined how the normal strain distribution was impacted by the OCA modulus properties. Each layer's normal strain distribution moved in the direction along which the strain gradually reduced as the modulus of OCA decreased. For instance, the normal strain shifted to the left when the OCA’s modulus decreased in the cover window of Fig. 5c (please also refer to the Supplementary Video S2 on normal strain distributions during the rotations of rollable displays). It was found that the stable condition and multi neutral plane effects of OCAs can be successfully investigated using the non-linear elastic OCA model, in contrast to the case of simple linear elastic OCA model in Fig. 4. The viscoelastic property of the realistic OCA is soft and slippery properties and allow consideration of large deformation. However, in previous studies of rollable display^44,45^, OCA was just assumed to be a fully elastic model and OCA was simply considered to be as one of solid material without viscoelastic property at all. Thus, in rolling deformation analysis, the OCA did not slip well between rigid functional layers, even if the modulus of OCA was sufficiently low. Therefore, this non-viscoelastic OCA consideration would lead not accurate rollable display design and results. This is significantly contrast to the OCA model considering viscoelasticity of realistic rollable display. For example, at the θ = 45°, 135°, 225°, and 315°, the normal strain distributions of rigid functional layers were clearly different shown as in Fig. 4a (non-viscoelastic OCA model) and in Fig. 5a (viscoelastic OCA model). In contrast to the non-viscoelastic OCA model, it was clearly found that viscoelastic OCA model demonstrated uniform normal strain distributions for different rolling angle positions. Moreover, owing to the sufficient slippery deformation of multi-OCA layers, proper multi-neutral plane effects can be considered. Therefore, in contrast to the simple linear elastic OCA model, it was confirmed that the viscoelasticity of OCA in rolling deformation can effectively reduce the normal strain of rigid functional layers. In summary, it can be believed that the viscoelastic effect consideration of OCA is highly important to properly design and to investigate of rollable displays which requires multi-neutral planes to reduce the normal strain of rigid functional layers under rolling deformations.

### Characteristics of shear strain distributions based on non-linear elastic OCA model

**Figure 6:**
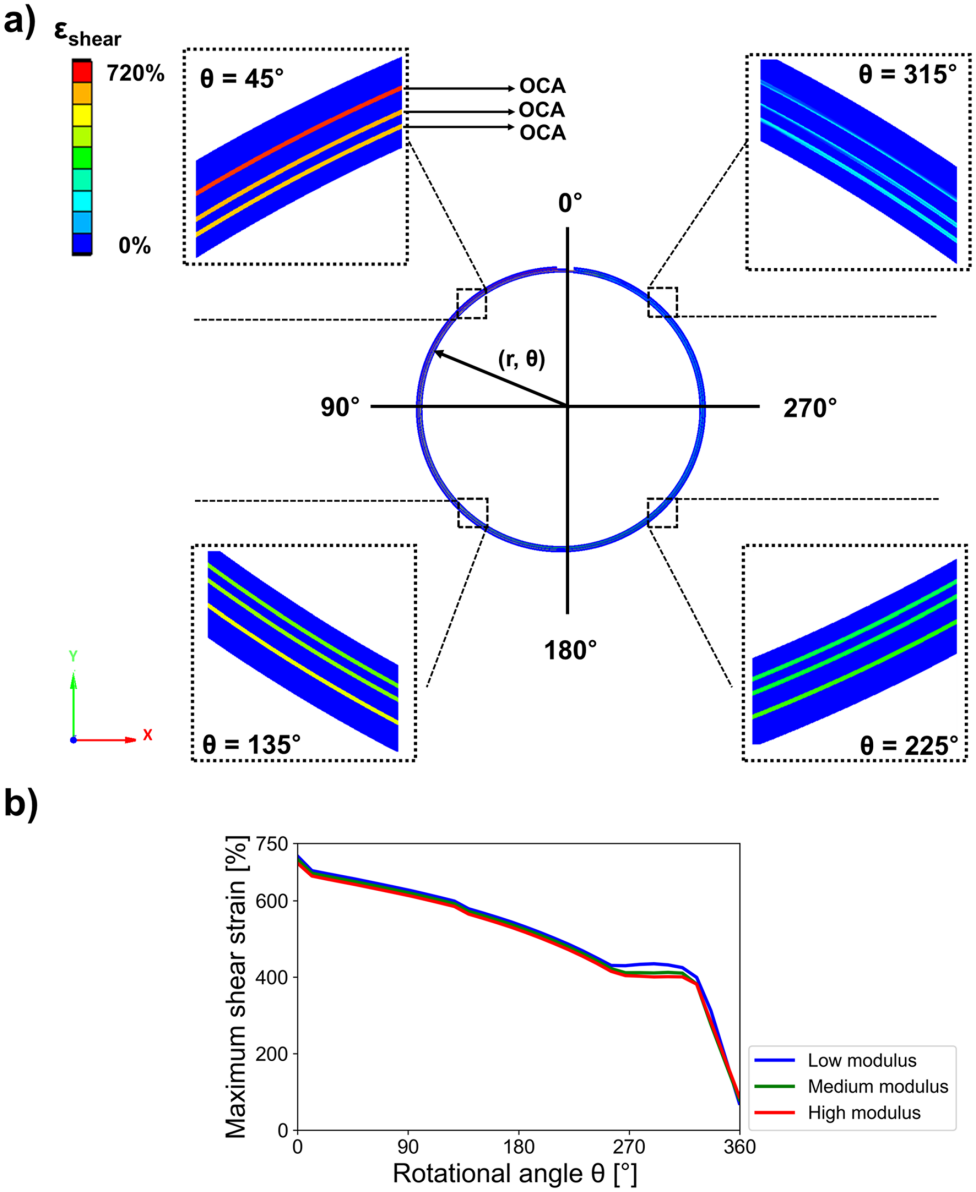
FEM results for shear strain in a rolling deformation case of a rollable display. (**a**) Shear strain distribution and enlarged FEM images. (**b**) Calculation of maximum shear strain applied to the cross-sections of OCA layers.

Figure 6a shows the shear strain FEM analysis results of the deformed, rolling display film stacks. The rigid functional layers generated a small-shear strain, whereas the OCA had a very high-shear strain. In this way, the shear strain of OCA inserted between the rigid functional layers caused a shear decoupling effect that reduced the normal strain applied to the display film stacks. Because all interfaces of the rigid functional layers received substantial shear decoupling effect from the OCAs during rolling deformation, we have previously verified the creation of multilayer neutral planes at 360°. The shear strain of OCA was calculated from all rotational angles, as shown in Fig. 6b. In a rolling deformation, the inner layers have a relatively small radius, and the outer layers have a relatively large radius. This difference in radius results in high shear deformation of OCA. Due to the geometrical characteristics of rolling deformation, it was found that the shear strain of OCA is decreased as the rotational angle gradually increased. Furthermore, the modulus of lower OCA increased the shear strain of OCA, and reduced the normal strain of the functional layers. The OCA modulus did not seem to have considerable influences on shear strain results because these low-, medium-, and high-modulus values were already sufficiently small. Nevertheless, when the OCA modulus was lower, it was assumed that the rollable display would be able to behave elastically farther from the permanent deformation region (please also refer to the Supplementary Video S3 on the shear strain distribution generated during the rotation of a rollable display).

## Conclusion

In this study, the mechanical behaviors of the multistacked rollable display were investigated based on considerations of the nonlinear properties of OCA adhesive interfaces. The multilayered structures of rollable displays, mechanical boundary conditions, and material modeling of OCA were described analytically. The FEM results for maximum normal strain in the rolling deformation were also analyzed as a function of the rotation angle. These results were compared analytically with the yield strain of each rigid functional layer, and showed that the rollable display was stable in the elastic region. In addition, the analysis findings for linear elastic OCA model were quantitatively compared in order to emphasize the rollable display FEM results of realistic nonlinear elastic OCA model. In 360° rotations considering a nonlinear elastic OCA model, the most vulnerable condition for strain, the normal strain distribution for rigid functional layer, and the shear strain distribution for OCA were estimated successfully. We found that shear and normal strains influenced each other at all positions. Moreover, the impact of the OCA's modulus on the distribution of shear and normal strain were also investigated. Accordingly, we demonstrated that the rollable display can exhibit increasingly stable behaviors against rolling mechanical deformation failure based on the confirmation that the reduction of OCA modulus increased shear but decreased normal strain. The FEM analyses of rollable displays, including the nonlinear elastic OCA and strain distribution information, conducted in this study, could be utilized for the mechanical analysis of various deformable electronic devices based on the use of soft adhesive interfaces.

## Supplementary Information

Supplementary Information 1.

Supplementary Video 1.

Supplementary Video 2.

Supplementary Video 3.

## Data Availability

Data underlying the results presented in this paper are available from the corresponding author upon reasonable request.
