# Supplementary material for: Analytical investigation of multi-layered rollable displays considering nonlinear elastic adhesive interfaces
Source: Sci Rep. 2023 Apr 7;13:5697. doi: 10.1038/s41598-023-31936-7 (PMC10082164; doi:10.1038/s41598-023-31936-7)
Supplement: Supplementary file 1 — Supplementary Information 1. [file 41598_2023_31936_MOESM1_ESM.docx]

Supplementary Information

Analytical investigation of multi-layered rollable displays considering nonlinear elastic adhesive interfaces

# Sang Hyun Han1, Jun Hyuk Shin1, and Su Seok Choi1,*

1Pohang University of Science and Technology (POSTECH), Department of Electrical Engineering, Pohang, 37673, Republic of Korea

* E-mail address

[choiss@postech.ac.kr](mailto:choiss@postech.ac.kr)

#
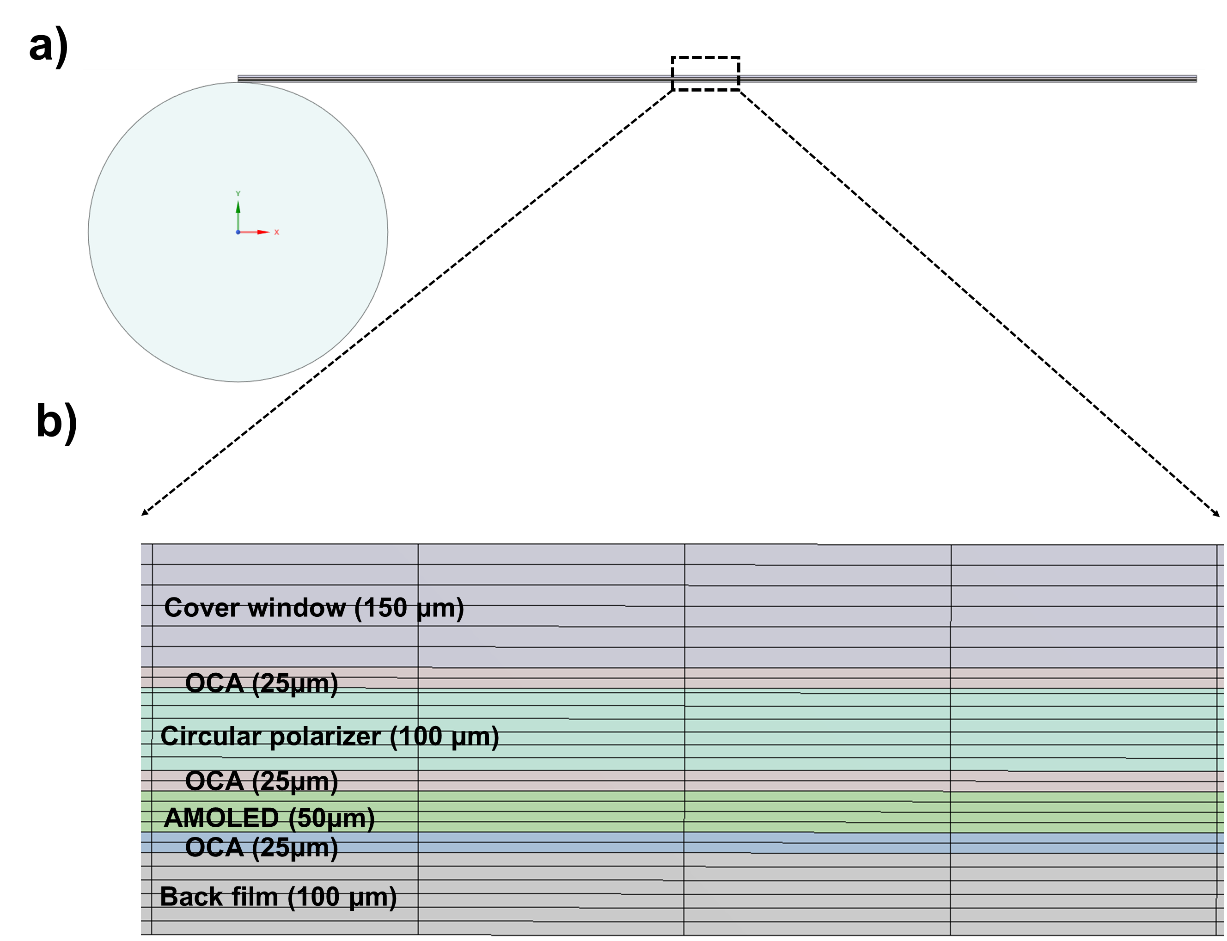


**Figure S1.** (a) FEM image for rollable display configuration. (b) Setting for the cross-sectional analysis of the film stacks and mesh configuration for FEM.

#
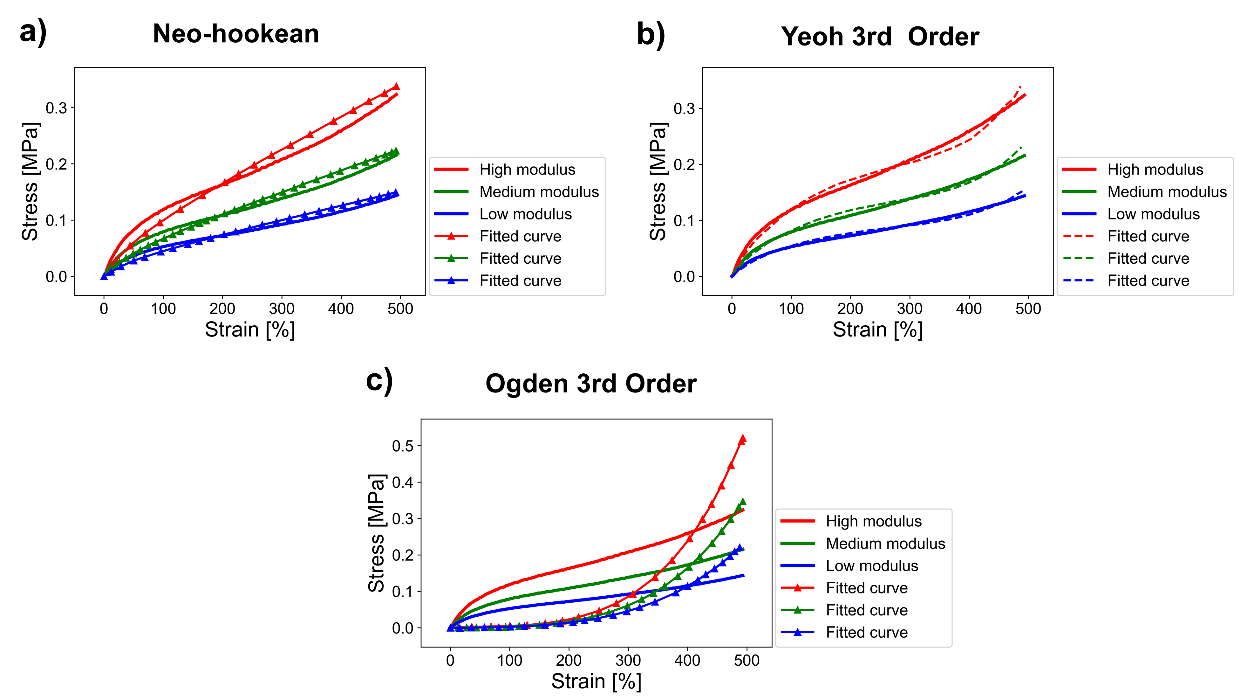


**Figure S2.** (a) Curve fitting results of OCAs using Neo-Hookean model. (b) Curve fitting results of OCAs using Yeoh 3rd order model. (c) Curve fitting results of OCAs using Ogden 3rd order model.


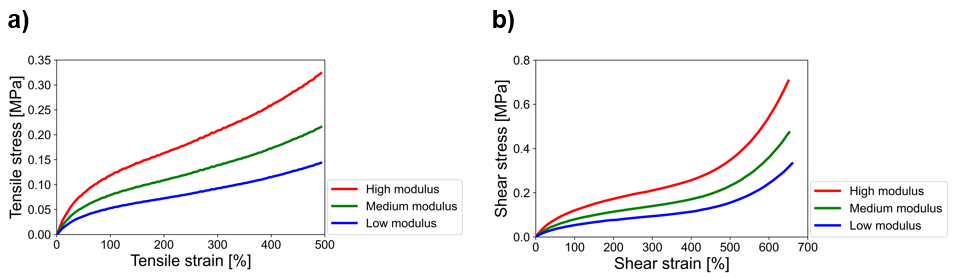


**Figure S3.** (a) Tensile stress-strain curve using Yeoh 3rd order model. (b) Shear stress-strain curve using Yeoh 3rd order model.

**Supporting Videos**

**Video S1**

Stress relaxation test of OCA with 20% constant uniaxial stretching for 10 seconds.

**Video S2**

Normal strain distribution of the rollable display during rolling deformation in Finite Element Method

**Video S3**

Shear strain distribution of the rollable display during rolling deformation in Finite Element Method
